# Impact of heparanase on renal fibrosis

**DOI:** 10.1186/s12967-015-0538-5

**Published:** 2015-06-04

**Authors:** Valentina Masola, Gianluigi Zaza, Maurizio Onisto, Antonio Lupo, Giovanni Gambaro

**Affiliations:** Renal Unit, Department of Medicine, Verona University Hospital, Piazzale A. Stefani 1, 37126 Verona, VR Italy; Department of Biomedical Sciences, University of Padova, Padua, Italy; Division of Nephrology and Dialysis, Columbus-Gemelli Hospital Catholic University School of Medicine, Rome, Italy

## Abstract

Tubulo-interstitial fibrosis has been recognized as the hallmark of progression of chronic kidney disease, but, despite intensive research studies, there are currently no biomarkers or effective treatments for this condition. In this context, a promising candidate could be heparanase-1 (HPSE), an endoglycosidase that cleaves heparan sulfate chains and thus takes part in extracellular matrix remodeling. As largely described, it has a central role in the pathogenesis of cancer and inflammation, and it participates in the complex biological machinery involved in the onset of different renal proteinuric diseases (e.g., diabetic nephropathy, glomerulonephritis). Additionally, HPSE may significantly influence the progression of chronic kidney damage trough its major role in the biological pathway of renal fibrogenesis. Here, we briefly summarize data supporting the role of HPSE in renal damage, focusing on recent evidences that demonstrate the capability of this enzyme to modulate the signaling of pro-fibrotic factors such as FGF-2 and TGF-β and consequently to control the epithelial-mesenchymal transition in renal tubular cells. We also emphasize the need of the research community to undertake studies and clinical trials to assess the potential clinical employment of this enzyme as diagnostic and prognostic tool and/or its role as therapeutic target for new pharmacological interventions.

## Background

Although improvements in drug therapies over the years have significantly slowed the progression of chronic kidney disease (CKD) to end-stage renal disease (ESRD), we still remain far from being able to prevent a sizable proportion of patients from ultimately need for dialysis or transplantation [1]. Therefore, because of the large worldwide diffusion of this clinical condition, this represents an important issue in medicine.

The US Renal Data System’s 2013 Annual Data Report indicates that more than 10% of the US adult population suffers from CKD [2]. In Italy, 13% of people over age 40 have CKD [3]. CKD and ESRD have a significant impact on health care costs. In the US, the cost of CKD patients over age 65 exceeds $45 billion [2].

Clinical-pathological studies have clearly shown that tubulo-interstitial fibrosis (TIF) is pivotally involved in the onset and progression of chronic renal damage and it could be considered a reliable prognostic marker of CKD, regardless of the etiology and the origin of the primary disease [4].

However, at the moment, main causes of renal fibrosis in developed countries are type 2 diabetes mellitus and ischemic/hypertensive nephropathy [5].

Additionally, despite numerous research efforts [6], no effective treatments or biomarkers are currently available for the progression of renal fibrosis. One interesting molecule that could be employed both as a biomarker and a pharmacological target is heparanase-1 (HPSE) an enzyme involved in the pathogenesis of several nephropathies [7] that cleaves heparan sulfate (HS) and thus takes part in the remodeling of the extracellular matrix (ECM) [8], a complex structure that acts as a scaffold for cell attachment, proliferation and differentiation, and modulates cell signaling [9].

The renal ECM consists of specific, specialized structures as the tubular and peritubular capillary basement membranes, and the glomerular basement membrane, but it also exists as an “amorphous” mesangial and interstitial matrix [10]. ECM remodeling by HPSE thus lies at the hub of networks integrating different mechanisms in renal fibrosis.

## Mechanisms of renal fibrosis

During renal fibrosis, the accumulation of abnormal ECM alters the three-dimensional structure of all the renal structures (glomeruli, tubules, interstitium and vasculature) and it induces a failure of the physiological mechanisms of response to acute insult and/or tissue regeneration. Several factors could be involved in this deregulated machinery (e.g., cytokines, ROS, or inflammatory cells) [11].

The main macroscopic features of renal fibrosis (glomerulosclerosis, TIF, inflammatory infiltration, and loss of renal parenchyma, characterized by tubular atrophy, capillary loss, and podocyte depletion) are caused by several biological events including mesangial and fibroblast activation, monocyte/macrophage and T cell infiltration, and cell apoptosis. The accumulation of connective tissue and infiltration of inflammatory cells and myofibroblasts into the renal parenchyma result in irreversible organ damage [11]. Activated myofibroblasts in the tubular compartment and mesangial cells in the glomeruli are key producers of ECM (collagen IV, laminin, fibronectin) and major responsible for TIF and glomerulosclerosis, respectively. Both these cell lines have also properties similar to contractile smooth muscle, since they express α-smooth muscle actin (α-SMA) [11]. In the kidney, myofibroblasts originate from several sources: (1) interstitial renal fibroblasts; (2) interstitial perivascular cells called pericytes; (3) fibrocytes; (4) tubular epithelial cells; and (5) endothelial cells [6, 12].

**Figure 1:**
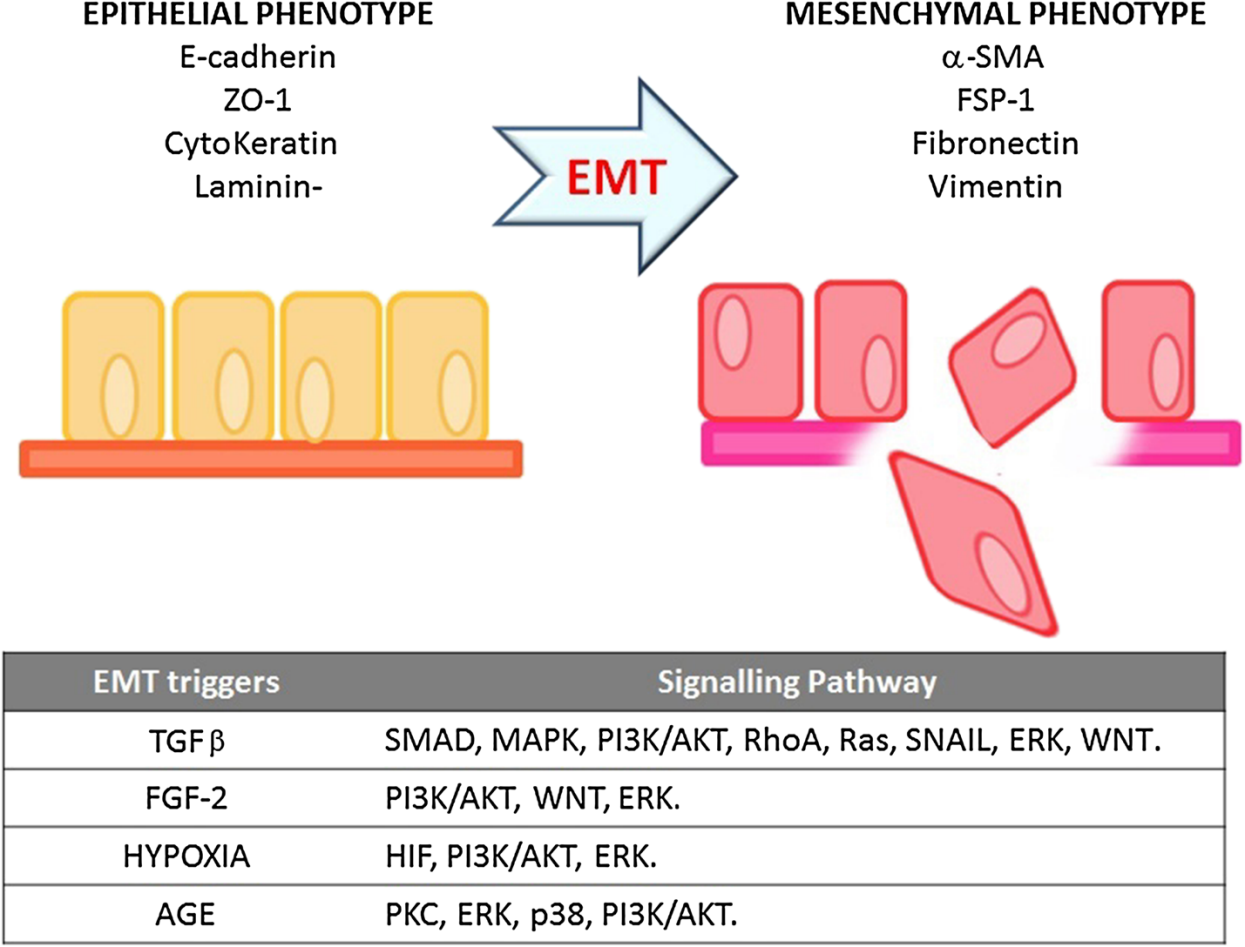
EMT process and signaling pathway. During EMT, tubular epithelial cells turn off the expression of adhesion molecules (E-cadherin, claudins and cytokeratins), and upregulate mesenchymal markers such as vimentin (VIM), fibronectin (FN), α-SMA, and fibroblast-specific protein-1 (FSP1). Subsequently, they undergo a reorganization of the cytoskeleton and a change in their morphology. The transition process from epithelial to mesenchymal phenotype is gradual and it involves intermediate morphological changes [14]. Degradation of the tubular basement membrane and migration of the transformed epithelial cells into the interstitium complete the EMT. The table at the bottom summarizes the main EMT triggers and the signaling pathways that they activate.

Tubular epithelial-mesenchymal transdifferentiation (EMT) into myofibroblasts, is a process first identified in mice and, more recently, in humans, representing an important source of these cells in kidney, contributes to the onset of TIF (Figure 1: top) and to the progression of CKD [13]. During this process, which ultimately determine renal fibrosis, the interstitial microenvironment is enriched with cytokines and chemokines released by the tubular epithelial cells in response to proteinuria, high glucose concentrations, advanced glycosylation end products (AGE), reactive oxygen species and hypoxia. Macrophages and lymphocytes attracted by the chemokines release additional factors, such as transforming growth factor-β (TGF-β), epidermal growth factor (EGF), and fibroblast growth factor-2 (FGF-2) [14]. All these stimuli contribute to EMT of tubular epithelial cells.

In particular, TGF-β seems to be primarily involved in the onset and progression of renal fibrosis in diabetic nephropathy (DN) [15]. In this pathological condition, the excess AGE and albumin filtering through the glomeruli also promotes EMT by directly stimulating the tubular cells to produce TGF-β [16].

Many other factors have been identified as EMT triggers and, together with TGF-β, they activate multiple and, in some cases, “redundant” signaling pathways such as SMAD, MAPK, PI3-K/Akt, RhoA [17–19] (Figure 1: bottom).

## Heparanase

**Figure 2:**
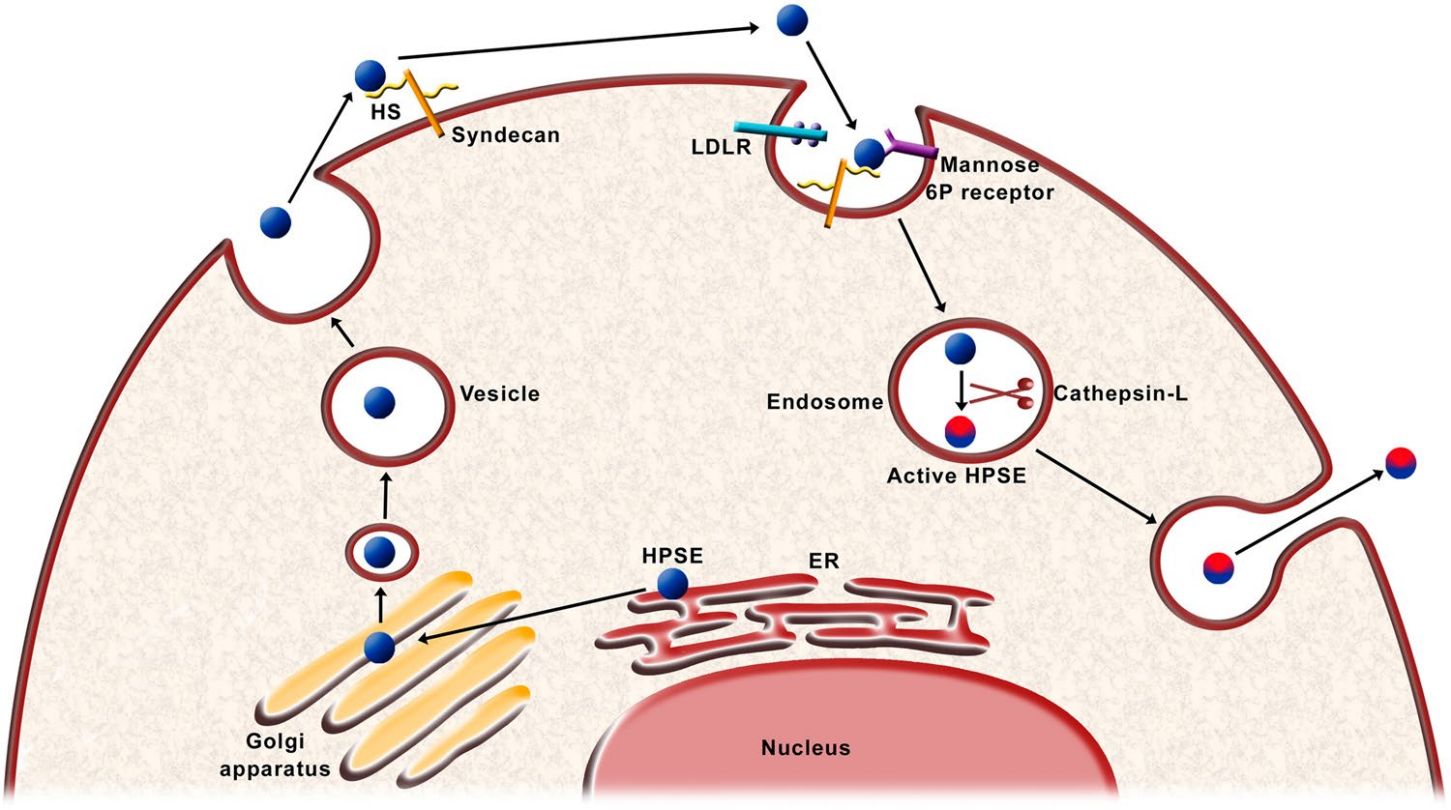
Graphical representation of HPSE maturation. The pro-enzyme is secreted from the Golgi complex. In the extracellular space, it interacts with syndecans or other receptors that mediate its endocytosis. The pro-enzyme is activated into lysosomes by cathepsin-l. Then active HPSE is secreted into the extracellular milieu.

HPSE is an endo-β (1-4)-d-glucuronidase that cleaves the β-(1,4)-glycoside bonds on the HS of HS-proteoglycans (HSPGs) leading to the production of HS fragments approximately 5–10 kDa [19]. The active form of this enzyme is a heterodimer consisting of a 50 kDa subunit associated non-covalently with an 8 kDa subunit [20]. The HPSE gene (50 kb) is on chromosome 4q21.3 and comprises 14 exons and 13 introns coding a precursor (pre-HPSE) that is post-translationally converted first into pro-HPSE and then into mature HPSE [19]. The inactive pro-HPSE (65 kDa) is transferred to the Golgi apparatus, where it is packaged in vesicles to be secreted from the cell. Once in the extracellular space, pro-HPSE interacts with syndecans and the pro-HPSE/syndecan complex is endocytosed [20]. Pro-HPSE can also be internalized by low-density lipoprotein receptor-related proteins and mannose 6-phosphate receptors [21]. Once inside the lysosomes, cathepsin-l cleaves the linker peptide from the pro-HPSE, yielding an heterodimer composed of 8- and 50-kDa subunits, thus activating HPSE (Figure 2).

Given its ability to degrade the HS chains, the main physiological function of heparanase is to regulate HS turnover and then participate in the remodeling of the ECM and basement membranes [20]. Nevertheless, HPSE triggers a number of signaling pathways by: (1) digesting HS chains and causing the release of growth factors and cytokines bound to HS, thereby regulating their free levels in the pericellular microenvironment; (2) interacting via its C-terminal domain [22], with several transmembrane heparanase-binding proteins (HBP) and activating kinases such as Src, Akt, and p38 MAPK; (3) modulating the activity of the FGF-2 and TGF-β signaling cascades [23, 24] and (4) converting the soluble HS fragments of SDC1 from inhibitors into potent activators of FGF-2 signaling [25].

In non-pathological conditions, HPSE is produced by placental trophoblasts, blood-borne cells and keratinocytes [19]. Although the participation of HPSE in a number of physiological processes (e.g., embryo implantation and development, hair growth, and inflammatory processes) [26], researchers nowadays are mainly focusing on its involvement in pathological conditions including tumor progression and renal diseases.

## Heparanase and renal fibrosis

Several studies of proteinuric renal diseases performed in animal models (puromycin amino nucleoside-induced nephrosis, streptozotocin-induced diabetic nephropathy and adriamycin nephropathy) and humans (diabetic nephropathy, membranous glomerulonephritis, IgA nephropathy, minimal change disease and dense deposit disease) have found a significant HPSE over-expression in the kidney tissue [27–29].

Additionally, since HPSE expression correlates inversely with HS content in the glomerular basement membrane, it has been suggested that the degradation of the glomerular HS by HPSE plays a role in the development of proteinuria [28, 30] particularly in diabetic nephropathy [7, 27, 29]. HPSE, constitutively expressed in human tubular epithelial cells, has also a physiological role in maintaining cellular and ECM homeostasis [31].

Changes in aforementioned balance, at least, partially determined by an abnormal HPSE regulation/activity, induce pathogenic mechanisms responsible for the onset of chronic kidney damage. In fact, in a model of type 1 diabetes induced by streptozotocin, HPSE-ko mice fail to develop chronic nephropathy [32] because protected against the onset of proteinuria and TIF [32].

HPSE expression is upregulated by transcription factors such as Sp1, GA-binding protein (GABP), ETS1, ETS2, early growth response-1 (EGR1), and downregulated by the transcription factor p53, and by DNA methylation. HPSE is also modulated by a variety of endogenous molecules: elastase, tissue plasminogen activator, thrombin, high glucose levels, angiotensin II and aldosterone, free radicals, pro-inflammatory cytokines, fatty acids, tissue necrosis factor-α (TNF-α), vascular endothelial growth factor (VEGF), and estrogens [7, 19].

**Figure 3:**
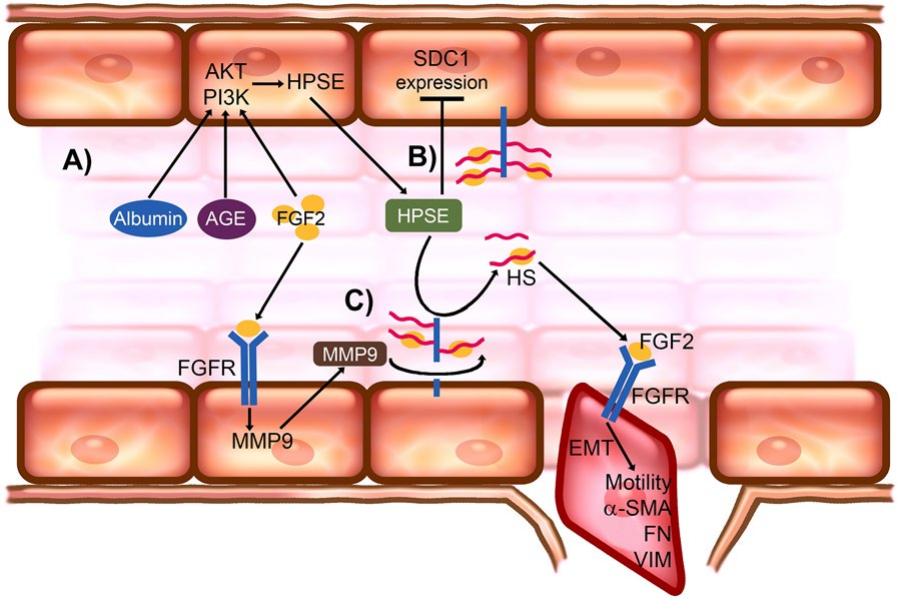
HPSE regulation and control of EMT induced by FGF-2. In the epithelial renal tubular cells (*A*), pro-fibrotic factors (albuminuria, AGE, and FGF-2) upregulate HPSE expression by activating PI3K/AKT; *B* HPSE overexpression triggers HS degradation and modulates syndecan-1 (SDC1) expression; *C* soluble HS fragments of SDC1 are potent FGF-2 signaling activators and thus facilitate tubular cell EMT.

Moreover, several pro-fibrotic factors such as albuminuria, high glucose concentrations, advanced glycosylation end products (AGE), TGF-β and FGF-2 can upregulate HPSE expression [23, 31] (Figure 3A) by activating PI3K/AKT [23, 31], a signaling pathway involved in the EMT of renal tubular cells [33]. The overexpression of HPSE can trigger HS degradation and modulate the expression of syndecan-1 (SDC1), the most abundant HSPGs on epithelial cells (Figure 3B). In this condition, the abnormal remodeling of HS and SDC1 can increase the availability of growth factors (e.g., FGF-2 and TGF-β leading to TIF).

Our in vitro data indicated that renal tubular cells lacking HPSE did not undergo EMT induced by FGF-2 and TGF-β [23]. This was mainly due to an over-expression of SDC1 on HPSE-silenced cells surface that has low affinity for FGF-2. In tubular cells lacking HPSE, the secondary impairment of PI3K/AKT pathway also jeopardizes the EMT induced by FGF-2. In addition, since FGF-2 is unable to increase the expression and activity of HPSE in such HPSE-deficient cells, the conversion of the soluble HS fragments of SDC1 from inhibitors into potent activators of FGF-2 signaling is prevented [23] (Figure 3C). Overall, HPSE is needed for FGF-2 to induce EMT in tubular cells, and to sustain FGF-2 signaling.

HPSE regulates TGF-β-related EMT [34], as well as its production. In fact, diabetic HPSE-ko mice, contrarily to the wild-type, did not show TGF-β increment in renal tissue and did not develop proteinuria, mesangial matrix expansion or TIF [32].

As demonstrated by our group, this effect could be partially explained by the fact that pro-fibrotic factors (albumin, AGE and FGF-2) need HPSE in order to upregulate TGF-β expression in renal tubular cells. Additionally, a lack of HPSE delays the onset of EMT induced by TGF-β and hampers the autocrine loop induced by TGF-β [24].

Another way in which HPSE may contribute to the progression of renal damage lies in its effect to regulate inflammation [35]. This effect has been reported in other chronic diseases [36, 37].

**Figure 4:**
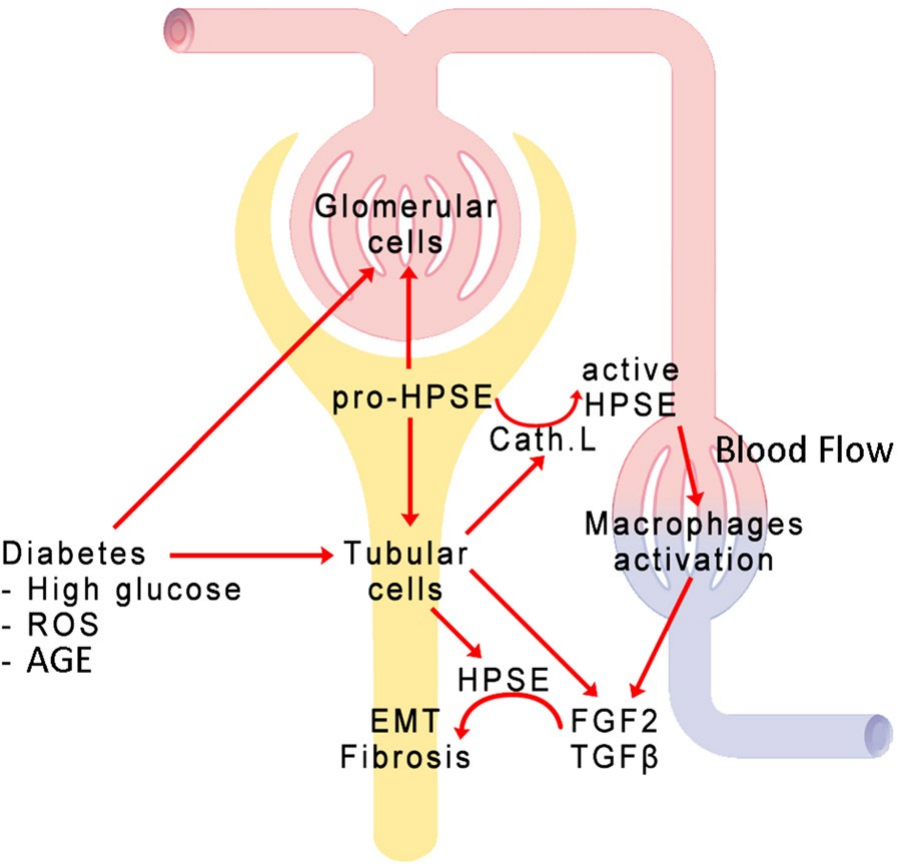
Involvement of HPSE in renal fibrosis. Several factors (high glucose concentrations, ROS, AGE) can increase HPSE production in both glomerular and tubular cells. The same factors induce cathepsin-l production by renal tubular cells, and this activates HPSE. Then the active HPSE can sustain inflammation by activating macrophages recruited from the circulation. During stress conditions both tubular and inflammatory cells release FGF-2 and TGF-β that, thanks to the higher amounts of HPSE, may activate the intracellular machinery leading to EMT.

In a recent paper, Gil et al. reported that HPSE-ko mice showed no significant infiltration of F4/80-positive macrophages in the renal parenchyma after the induction of type 1 diabetes, unlike wild-type animals [32]. Goldberg et al. [38] have also recently shown that HPSE fuels chronic inflammation in diabetic nephropathy: inactive HPSE produced by glomeruli and activated by cathepsin-l released by tubular cells sustains a persistent activation of kidney-damaging macrophages, thus creating chronic inflammatory conditions and fostering macrophage-mediated renal injury (Figure 4).

Overall, the ability of HPSE to regulate multiple mechanisms (glomerular permselectivity, tubular cell EMT, inflammation) makes it a central player in the fibrogenic process of diabetic nephropathy and, since similar mechanisms occur in other chronic kidney diseases (including chronic allograft dysfunction), it could be an effective therapeutic target.

## Heparanase as a biomarker

Heparanase has been detected in the plasma of neoplastic patients and its expression has been correlated with a better survival rate [39–41].

Increased urine and plasma levels of HPSE have been, also, reported in patients affected by a number of renal diseases (mainly diabetic nephropathy). In diabetes, HPSE levels correlated with blood glucose levels [42, 43] and, because involved in the atherosclerotic plaque development, it has been proposed as biomarker to early identify patients with DN at high risk to develop cardiovascular complications [44]. In kidney transplant recipients, plasma HPSE levels were associated with proteinuria and renal allograft function impairment [45].

Furthermore, our unpublished data, obtained by analyzing several chronic kidney diseases, suggest that HPSE levels are inversely associated with the degree of renal function impairment.

Cohen-Mazor et al. [46] reported that plasma levels of HPSE were higher in hemodialysis patients compared to healthy subjects probably due to degranulation of primed circulating PBMC during the dialysis session. This was later confirmed by our findings showing that HPSE expression in peripheral blood mononuclear cells (PBMC) and plasma were significantly up-regulated in both peritoneal- and hemo-dialysis treated patients [47]. The significant correlation between HPSE plasma activity and C-reactive protein levels also demonstrated a close involvement of the microinflammation in the proteoglycan metabolism deregulation in uremic patients [47].

However, future investigations are necessary to elucidate whether urinary or plasma HPSE levels could serve as biomarkers of the onset and progression of fibrosis.

## Heparanase as pharmacological target

In the last years, a number of HPSE inhibitors have been developed and some of them have entered clinical trials for cancer [48–50]. They include heparan sulfate mimetics, heparin-derived compounds, or other oligosaccharides that compete with the heparan sulfate chain for binding to heparanase [49]. Glycosaminoglycans such as sulodexide are effective HPSE inhibitors [51] and consequently have potential as drugs for the treatment of CKD and preventing progression to renal failure [52].

Important evidence of the utility of anti-HPSE therapies to control the progression of renal fibrosis comes from both in vitro and in vivo studies. In tubular cells, HPSE inhibition prevents the HS degradation induced by pro-fibrotic factors [31], EMT induced by FGF-2 [23, 51], and TGF-β upregulation after treatment with pro-fibrotic factors [24]. In animal models, a beneficial effect on the onset and development of diabetic nephropathy, and on the renal overexpression of TGF-β has been obtained with heparin-derived drugs [53], and sulodexide [54]. This issue was specifically investigated in mouse models of DN using the specific heparanase inhibitor SST0001, which markedly reduced the extent of albuminuria and renal damage [32].

Moreover, targeting HPSE in CKD could be useful for reducing the risk of cardiovascular complications [55]. Sulodexide seems to have some interesting favorable effects in the prevention and treatment of the complications of atherosclerosis, possibly supporting its role as HPSE inhibitor [56, 57].

## Conclusion

Several studies revealed that HPSE plays a pivotal role in the pathogenesis of a great number of renal diseases (e.g., diabetic nephropathy, chronic allograft nephropathy) and in the progression of chronic renal damage.

However, at the moment, we are far from an employment of HPSE measurement in the “day by day” clinical practice and it is unquestionable that additional studies and clinical trials should be undertaken to assess its potential employment as diagnostic and prognostic tool and/or therapeutic target for new pharmacological interventions.

## Abbreviations

AGE: advanced glycosylation end products
CKD: chronic kidney disease
DN: diabetic nephropathy
ESRD: end-stage renal disease
EGF: epidermal growth factor
EMT: epithelial-mesenchymal transdifferentiation
ECM: extracellular matrix
FGF-2: fibroblast growth factor-2
HPSE: heparanase-1
HS: heparan sulfate
HSPGs: HS-proteoglycans
PBMC: peripheral blood mononuclear cell
SDC1: syndecan-1
TNF-α: tissue necrosis factor-α
TLRs: toll-like receptors
TGF-β: transforming growth factor-β
TIF: tubulo-interstitial fibrosis
VEGF: vascular endothelial growth factor
